# The Immunogenicity of Capsid-Like Particle Vaccines in Combination with Different Adjuvants Using Different Routes of Administration

**DOI:** 10.3390/vaccines9020131

**Published:** 2021-02-06

**Authors:** Christoph M. Janitzek, Philip H. R. Carlsen, Susan Thrane, Vijansh M. Khanna, Virginie Jakob, Christophe Barnier-Quer, Nicolas Collin, Thor G. Theander, Ali Salanti, Morten A. Nielsen, Adam F. Sander

**Affiliations:** 1 Centre for Medical Parasitology, Department of Immunology and Microbiology, University of Copenhagen, 1165 København, Denmark; rdx520@alumni.ku.dk (C.M.J.); phrc@dtu.dk (P.H.R.C.); susanthrane@gmail.com (S.T.); vijanshpr@gmail.com (V.M.K.); thor@sund.ku.dk (T.G.T.); salanti@sund.ku.dk (A.S.); mortenn@sund.ku.dk (M.A.N.); 2 Department of Infectious Diseases, Copenhagen University Hospital, 2100 Copenhagen, Denmark; 3 Vaccine Formulation Institute, Plan-Les-Ouates, 1228 Geneva, Switzerland; virginie.jakob@vformulation.org; 4 Vaccine Formulation Laboratory, University of Lausanne, 1015 Lausanne, Switzerland; barnierquer@gmail.com (C.B.-Q.); nicolas.collin@unil.ch (N.C.)

**Keywords:** vaccine, capsid-like particle, virus-like particle, adjuvants, AP205, route of immunization

## Abstract

Capsid-like particle (CLP) displays can be used to enhance the immunogenicity of vaccine antigens, but a better understanding of how CLP vaccines are best formulated and delivered is needed. This study compared the humoral immune responses in mice elicited against two different vaccine antigens (a bacterial protein and a viral peptide) delivered on an AP205 CLP platform using six different adjuvant formulations. In comparison to antibody responses obtained after immunization with the unadjuvanted CLP vaccine, three of the adjuvant systems (neutral liposomes/monophosphoryl lipid A/quillaja saponaria 21, squalene-in-water emulsion, and monophosphoryl lipid A) caused significantly increased antibody levels, whereas formulation with the three other adjuvants (aluminum hydroxide, cationic liposomes, and cationic microparticles) resulted in similar or even decreased antibody responses. When delivering the soluble bacterial protein in a squalene-in-water emulsion, 4-log lower IgG levels were obtained compared to when the protein was delivered on CLPs without the adjuvant. The AP205 CLP platform promoted induction of both IgG1 and IgG2 subclasses, which could be skewed towards a higher production of IgG1 (aluminum hydroxide). Compared to other routes, intramuscular administration elicited the highest IgG levels. These results indicate that the effect of the external adjuvant does not always synergize with the adjuvant effect of the CLP display, which underscores the need for empirical testing of different extrinsic adjuvants.

## 1. Introduction

Recombinant protein expression holds promise in enabling the production of effective vaccines against many diseases [1], yet soluble proteins often fail to produce sufficient immune protection due to intrinsic low immunogenicity [1,2]. To overcome suboptimal immunogenicity of subunit vaccines, a number of novel adjuvant systems have been developed to boost or modulate immune responses induced by vaccine antigens [3]. Capsid-like particles (CLPs) are promising tools for vaccine development. CLPs are highly immunogenic due to their structural resemblance to live viruses, particularly their size (20 nm–200 nm) allows for direct drainage into lymph nodes [4,5]. In addition, the repetitive surface structure promotes uptake and cross presentation by antigen-presenting cells [6,7,8] and facilitates efficient B cell receptor crosslinking [9,10]. CLPs can be used as scaffolds for the presentation of unrelated antigens. CLP display technologies exploit the delivery of an antigen in a particulate, multivalent, and repetitive format increasing the immune-stimulatory activity of the antigen. In fact, many modular CLP-based vaccine platforms have emerged [11,12,13,14,15,16,17,18] and make it possible to employ a common CLP backbone for delivery of a vast variety of vaccine antigens [19,20,21,22,23].

Our group previously developed a modular CLP-based vaccine platform using the AP205 bacteriophage CLP as a backbone for covalently attaching diverse antigens on their surface through a tag/catcher split-protein interaction [14]. As this CLP platform is becoming a generic tool for vaccine development, we sought to explore whether formulating the CLP in extrinsic adjuvants [24,25] affects the humeral immune response to the vaccine and whether a particular immunization route is preferable for vaccine administration.

These factors might be dependent on the specific antigen being presented on the CLP. Nonetheless, in this study, we used the SpyCatcher-AP205-L2 vaccine [19] as a prototype AP205 CLP vaccine displaying both a vaccine peptide (Human Papilloma Virus (HPV) 16 L2 RG1 epitope) and a poorly immunogenic bacterial protein (SpyCatcher). This dual-antigen CLP vaccine provided the basis for evaluating the humoral immune response in mice obtained by using different extrinsic adjuvant formulations that are either comparable to adjuvants already used in humans (neutral liposomes/monophosphoryl lipid A/quillaja saponaria 21 (LMQ), squalene-in-water emulsion (SWE), monophosphoryl lipid A (MPL), and aluminum hydroxide (AlOH)) or are promising experimental adjuvants (cationic liposomes (CL) and cationic microparticles (MP)) (Table 1). Lastly, we compared the immunogenicity of the CLP vaccine when using different immunization routes.

## 2. Materials and Methods

### 2.1. Design, Expression, and Purification of SpyCatcher-AP205-L2

SpyCatcher-AP205-L2 was designed, recombinantly expressed, and purified as described in [19]. Briefly, the RG1 epitope of the minor capsid protein (L2) of Human Papilloma Virus (HPV) 16 (QLYKTCKQAGTCPPDIIPKVEG) was attached by PCR amplification to the 3′ end of the SpyCatcher-AP205 construct [14]. The SpyCatcher-AP205-L2 construct was inserted into a pet-15b vector, expressed in One Shot^®^ BL21 Star™ (DE3) cells (Thermo Scientific), and purified on Optiprep™ (Sigma-Aldrich, Denmark) step gradients, as previously described [14,19,22]. Throughout this article, the above described SpyCatcher-AP205-L2 CLP vaccine is referred to as the “CLP vaccine”.

### 2.2. Design, Expression, and Purification of SpyCatcher and HPV16 L2 Control Antigens

To produce the recombinant SpyCatcher control antigen, a flexible Glycine-Glycine-Serine (GGS) linker, followed by a hexahistidine (6xHis)-tag was added to the C-terminus of SpyCatcher. SpyCatcher was produced in One Shot^®^ BL21 Star™ (DE3) cells (Thermo Scientific) and purified using ion metal affinity chromatography as well as ion exchange chromatography. This protein was used as the coat for anti-SpyCatcher IgG ELISA and as the control antigen in mouse immunizations (described below)

The RG1 epitope of HPV16 (QLYKTCKQAGTCPPDIIPKVEG) was attached by overlap extension PCR to the 5′ end of a biologically irrelevant carrier protein (accession number WP_057363222, amino acid 44-338). The HPV peptide/protein fusion was expressed in One Shot^®^ BL21 Star™ (DE3) (Thermo Scientific) and purified using ion metal affinity chromatography as well as size exclusion chromatography (HiLoad Superdex 75pg, GE Healthcare). This protein was used for coat protein in the anti-HPV peptide ELISAs.

### 2.3. Design, Expression, and Purification of SpyTag-MS2 and Preparation of SpyCatcher-MS2 

To raise the antibody titers against SpyCatcher and SpyTag in mice, a different CLP backbone than AP205 was required. For this reason, the MS2 CLP was used to make SpyTag and SpyCatcher presenting MS2. SpyTag-MS2 was designed to include the SpyTag (AHIVMVDAYKPTK) at the N-terminus of the MS2-CLP gene (PDB 1U1Y). This gene was subcloned into a pet-15b vector and transformed into One Shot^®^ BL21 Star™ (DE3) cells (Thermo Scientific) for expression. The assembled SpyTag-MS2 CLP was purified on Optiprep™ (Sigma) step gradients, as described for AP205.

SpyCatcher-MS2 CLP were created by incubating SpyTag-MS2 CLPs with SpyCatcher-ggs-His control antigen (production described above) at a 1 to 5 molar ratio for 16 h at 4 °C. Excess SpyCatcher was removed by dialyzing against 1 × phosphate buffered saline (PBS) using 1000 kDa molecular weight cut-off (MWCO) tubing (SpectraPor).

### 2.4. Removal of Endotoxins

Triton X-114 was used in order to remove endotoxins from the CLP vaccines (prototype AP205 CLP vaccine and the MS2 CLP vaccines used to raise preexisting antibodies) as well as control antigens, as described in [51].

### 2.5. Vaccine Formulation

The CLP vaccine was diluted to 0.1 µg/µL and formulated under sterile conditions 1 h prior to immunization in the following extrinsic adjuvant formulations: aluminum hydroxide (alhydrogel/AlOH), squalene water emulsion (SWE), a mix of neutral liposomes (DOPC–cholesterol)/monophosphoryl lipid-A/quillaja saponin 21 (QS21) (together called LMQ), cationic microparticles (MP), cationic liposomes (CL), or monophosphoryl lipid-A (MPL), as indicated in Table 2. The amount of each compound included in the different adjuvant formulations is shown (per dose of 50 µl) in Table 3. Control vaccines consisting of soluble SpyCatcher formulated in either LMQ or SWE were administered using a similar antigen dose (i.e., similar copy number of SpyCatcher) to that used for immunizations with the SpyCatcher CLP vaccines. The neutral liposomes, SWE, CL, and MP were manufactured at the Vaccine Formulation Laboratory. QS21 and MPL-A (via sonication in aqueous buffer) solutions were resuspended at the Vaccine Formulation Laboratory (1 mg/mL). LMQ adjuvants were mixed immediately prior to formulating with the CLP or the control antigen.

### 2.6. Mouse Immunization Studies

Adjuvant formulation study: In one study, female, 8-week-old BALB/c mice (Janvier Labs, France) were immunized twice (week 1 and 3) with 2 µg of CLP vaccine formulated in extrinsic adjuvants (as described above and in Table 2, n = 6 per adjuvants group) or without (n = 6). Mice were immunized intramuscularly (IM) in the thigh muscle (50 µL per dose). The IgG ELISA determination included n = 6 mice for the LMQ, SWE, MPL, and CL groups; n = 5 for the no adjuvant group and MP groups; and n = 4 or n = 5 for the AlOH group (anti-SpyCatcher or anti-HPV respectively).

#### Route of Immunization Study

In another study, female BALB/C mice (8 weeks of age) were also immunized with 2 µg of CLP vaccine formulated without extrinsic adjuvants and using alternative routes of injection (n = 6 per immunization route). Mice received 30 µL per dose using intradermal injections (ID, on the back, shaved prior to injection), subcutaneous injections (SC, in the neck fold), intramuscularly (in the thigh muscle), and intra nasally (IN). For immunizations into the peritoneal cavity (IP), 100 µL injection volume was used. Mice receiving intradermal, subcutaneous, and intranasal immunizations were briefly anesthetized using isoflurane.

For both abovementioned studies, blood samples were collected on week 8. Serum purified from these blood samples was used for antigen-specific IgG ELISA and IgG isotype subtyping.

### 2.7. Antigen-Specific Serum Immunoglobulin Levels

In order to detect the level of anti-SpyCatcher and anti-L2 (HPV16 RG1 epitope) IgG, following the immunization of mice with the CLP vaccine, a standard enzyme-linked immunosorbent assay (ELISA) was employed as described previously [19]. Briefly, to detect SpyCatcher-specific IgG titers, 96-well microtiter plates (Nunc MaxiSorp were incubated with 0.1 µg per well SpyCatcher-ggs-His or HPV16 L2 RG1 antigen; 1 × PBS buffer was used for all serum dilutions, and 1 × PBS + 0.5% skim milk powder (w/v) was used for blocking the plates. Secondary goat anti-mouse IgG-horseradish peroxidase (Novex) was used along with 3,3′,5,5′-Tetramethylbenzidine (TMB) as a substrate for developing the plates. The reaction was stopped with 0.2 M H_2_SO_4_, and the color signal was measured at O.D. 450 nm. A control sample containing high levels of antigen-specific IgG was used across all ELISA plates for normalization. The antibody titers were measured as area under the curve values for both SpyCatcher or HPV16 L2 IgG using an y-axis cutoff calculated as follows: mean (O.D. 450 nm of background wells) + 3× standard deviation (O.D. 450 nm of background wells).

### 2.8. Serum IgG Subclass Profiling by ELISA

The relative proportions of mouse IgG isotypes following immunization with the CLP vaccine formulated in different extrinsic adjuvants (or without) was determined by ELISA as described previously [14]. First, the IgG was normalized by dilution, according to a predetermined O.D. 450 nm value (i.e., obtained by a serum dilution in a standard ELISA described above). Hereafter, secondary HRP-conjugated antibodies targeting mouse total IgG (Novex), IgG1 (Invitrogen), IgG2a (Invitrogen), IgG2b (Thermo Fischer), or IgG3 (Thermo Fischer) were used for detection of the different subpopulations of antibody isotypes. Each serum sample was measured in triplicate and developed for 7 min with o-phenylenediamine substrate. The enzymatic reaction was stopped by adding of 2 M H_2_SO_4_. Optical density was measured at 490 nm. To determine the % IgG subclass of total IgG, the obtained O.D. 490 nm value measured following incubation with the antibodies targeting different IgG subclass was divided by the O.D. 490 nm value obtained by mouse total, SpyCatcher-specific, IgG (Novex). For the no adjuvant group and MP, n = 3; for the remaining groups, n = 6.

## 3. Results

**Figure 1:**
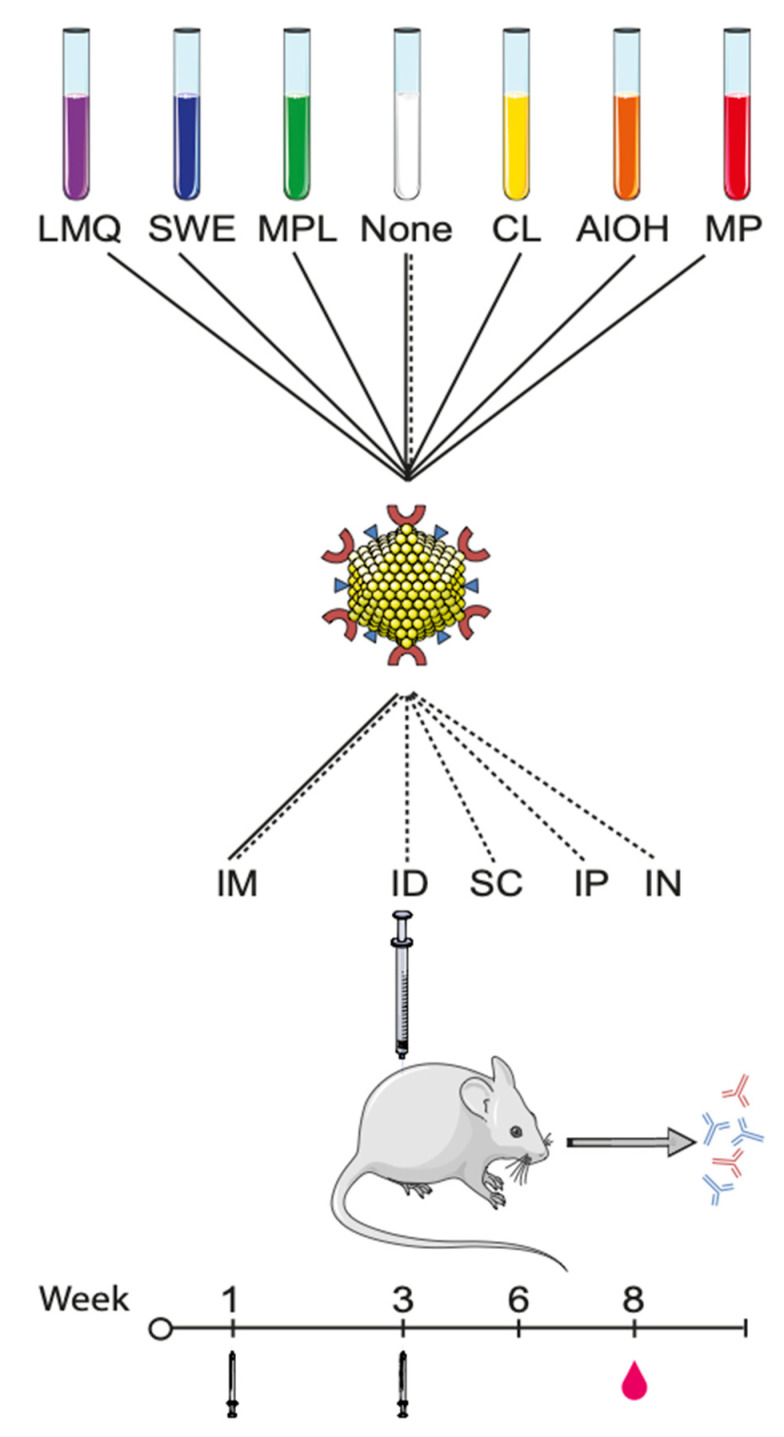
Schematic overview of the adjuvant formulations, vaccination routes, and timepoints employed: SpyCatcher-AP205-L2 CLP (SpyCatcher—red “C” shapes, L2 RG-1 peptide epitope—blue triangles, and CLP structural proteins—yellow spheres) were formulated in six extrinsic adjuvants, including liposomes/MPL/QS21 (LMQ, purple), squalene water emulsion (SWE, blue), monophosphoryl lipid A (MPL, green), cationic liposomes (CL, yellow), aluminum hydroxide (AlOH, orange), or Microparticles (MP, red) or without extrinsic adjuvants (none, white). The mice were immunized intramuscularly (IM) with all CLP formulations in a prime-boost regimen at weeks 1 and 3. Blood was drawn on week 8 (i.e., 5 weeks after last immunization). In a separate study, SpyCatcher-AP205-L2 was formulated without adjuvants (none) and immunized in a prime boost regiment via different routes of injections, specifically IM, intraperitoneally (IP), subcutaneously (SC), intradermally (ID), and intranasally (IN). The graphics of the mouse, the CLP, and reagent tubes have been modified from Servier Medical ART under a creative commons license (https://creativecommons.org/licenses/by/3.0/legalcode (accessed on 6 February 2021)).

The CLP-based vaccine, SpyCatcher-AP205-L2, was employed. This vaccine is based on recombinant expression in *E. coli* of an AP205 capsid protein containing an N-terminal SpyCatcher protein and a C-terminal tail of 23 peptides corresponding to the RG1 epitope of the L2 protein of Human Papilloma Virus (HPV) 16. These genetically modified structural AP205 proteins spontaneously form CLP, presenting the SpyCatcher protein and the HPV peptide on protrusion from their surface [19]. In this study, the SpyCatcher protein serves as a model vaccine antigen, although the intended function is to spontaneously form a covalent bond with a recombinant protein carrying a SpyTag. This allows for a simple conjugation reaction resulting in the formation of a covalent bond between two components, i.e., a protein vaccine antigen and the AP205 particle [19]. Groups of six mice were vaccinated twice, three weeks apart, with the CLP vaccine in phosphate buffered saline or in the presence of one of six adjuvants (see Figure 1 for an overview and Table 1 for details). As a control, two groups of mice were vaccinated with recombinant soluble SpyCatcher not displayed on CLP but formulated in LMQ or SWE.

### 3.1. Comparison of Humoral Responses Induced by Prototype CLP Vaccine Formulated with Different Extrinsic Adjuvants

**Figure 2:**
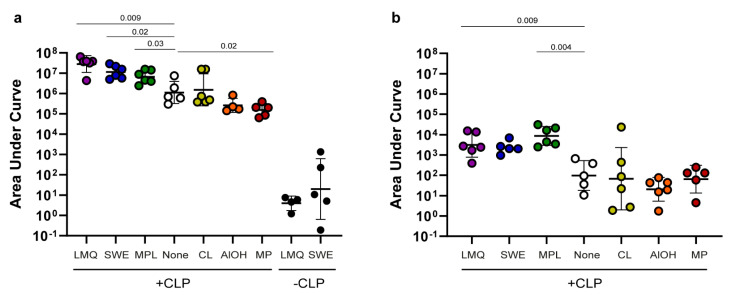
Immunogenicity of antigens on CLP in extrinsic adjuvant formulations: (**a**) SpyCatcher and (**b**) HPV peptide-specific IgG levels measured as the area under the curve (AUC) (AUC = the optical density (O.D.) at 450 nm multiplied by the dilution factor) following immunization with the SpyCatcher-AP205-L2 (+CLP) vaccine formulated in different adjuvants. Specifically, mice were immunized with liposomes/MPL/QS21 (LMQ, purple, n = 6), squalene water emulsion (SWE, blue, n = 6), monophosphoryl lipid A (MPL, green, n = 6), no adjuvants (none, white, n = 5), cationic liposomes (CL, yellow, n = 6), aluminum hydroxide (AlOH, orange, n = 4) or microparticles (MP, red, n = 5). The control groups were immunized with soluble SpyCatcher protein formulated in either LMQ or SWE (−CLP, black, n = 6 for both groups) using a similar antigen dose (i.e., similar copy number of SpyCatcher) to that used for the SpyCatcher CLP vaccine. Each circle represents the AUC antibody levels obtained in a single mouse in one representative ELISA assay measurement at an O.D. 450 nm cutoff of 0.1. The geometric mean is shown together with the geometric standard deviation. The Kruskal–Wallis test determined that there was a statistic significant difference between two or more groups ((**a**) SpyCatcher *p* < 0.0001 and (**b**) HPV peptide *p* = 0.0011). For SpyCatcher, Mann–Whitney nonparametric tests were used to compare the LMQ and SWE control vaccines (−CLP) with their respective LMQ and SWE (+CLP) test vaccines and found significantly increased levels (LMQ *p* = 0.0095 and SWE *p* = 0.0043). Hereafter, for both SpyCatcher and the HPV peptide, uncorrected, pairwise Mann–Whitney tests were used to compare the different +CLP adjuvant groups with the un-adjuvanted CLP. Only significant *p*-values in comparison to the non-adjuvanted group are presented in the graph. See Supplementary Figure S2 for all *p*-value calculations.

The responses were compared by measuring SpyCatcher protein or HPV peptide-specific immunoglobulin G (IgG) levels, measured as the area under the curve (AUC) in serum collected five weeks after the last immunization (Figure 1). The control vaccines (SpyCatcher without CLP display) adjuvanted with LMQ or SWE elicited limited or no anti-SpyCatcher IgG (Figure 2a). By contrast, displaying the antigen on the surface of the CLP and formulating in the same adjuvants resulted in significantly higher levels of anti-SpyCatcher IgG (7-fold log increase and 6-fold log increase for LMQ (*p* = 0.0095) and SWE (*p* = 0.0043), respectively), in all vaccinated animals. Administering the CLP vaccine with LMQ, SWE, or MPL significantly boosted anti-SpyCatcher IgG levels (*p* = 0.009, *p* = 0.02 and *p* = 0.03 for LMQ, SWE, and MPL, respectively) compared to the non-adjuvanted CLP vaccine (corresponding to about 1–1.5 log increase in endpoint titers, Supplementary Figure S1). Mice receiving the CLP vaccine with CL, AlOH, or MP had similar (CL and AlOH) or lower (MP, *p* = 0.02) antibody levels compared to mice receiving the CLP-based vaccine without adjuvant (this corresponds to a one-log drop in endpoint titer for MP, Supplementary Figure S1).

When comparing yjr IgG responses to the HPV peptide, similar results to that for the SpyCatcher CLP were seen (Figure 2b). Mice receiving the CLP vaccine adjuvanted with LMQ, SWE, or MPL had higher IgG AUC levels (corresponding to approximately one log higher endpoint titers, Supplementary Figure S1) against the HPV peptide compared to mice receiving the CLP vaccine without adjuvant. However, this trend was only significant for LMQ and MPL (*p* = 0.009 and 0.004 for LMQ and MPL, respectively). Likewise, for the anti-HPV peptide response, the mice receiving the CLP vaccine adjuvanted with CL, AlOH, or MP had similar antibody levels compared to mice receiving the CLP without adjuvant.

### 3.2. Induction of IgG Subtype Profiles by CLP in Different Adjuvant Formulations

**Figure 3:**
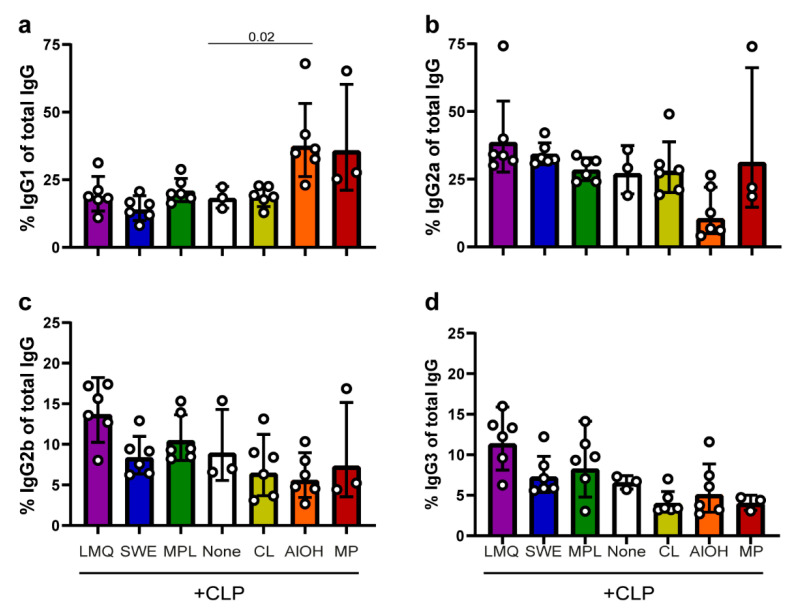
Characterization of vaccine-induced IgG subclasses. To characterize the IgG responses induced by the SpyCatcher-AP205-L2 vaccine formulated in the different extrinsic adjuvants, the relative proportions of IgG subclasses were normalized against the total vaccine-induced IgG. The four panels show the relative amounts of IgG1 (**a**), IgG2a (**b**), IgG2b (**c**) and IgG3 (**d**). The geometric mean is displayed along with the geometric standard deviation (SD). Kruskal–Wallis test showed that there was significant differences between two or more groups ((**a**) *p* = 0.0016, (**b**) *p* = 0.0085, (**c**) *p* = 0.0380, and (**d**) *p* = 0.0052). Hereafter, uncorrected, pairwise Mann–Whitney analysis was conducted to assess statistically significant differences between each of the different adjuvant groups and the un-adjuvanted group (none). Significant differences are indicated in the graph.

To further evaluate how the extrinsic adjuvants modify the immune response generated by the CLPs, the serum levels of (anti-SpyCatcher) IgG subclasses were measured eight weeks after the first immunization. Figure 3 shows the relative amount of IgG subclasses produced (i.e., normalized against the total level of anti-SpyCatcher IgG). Mice vaccinated with the CLP vaccine produce high relative levels of both IgG1 (Figure 3a) and IgG2a (Figure 3b) and to a lesser extent IgG2b (Figure 3c) and IgG3 (Figure 3d) (geometric means of 18%, 27%, 9%, and 6.5%, respectively). Administering the CLP in LMQ, SWE, MPL, CL, or MP does not alter the overall IgG subclass distribution produced by the CLP alone. However, mice vaccinated with CLP formulated in LMQ, SWE, or MPL had produced higher overall IgG levels and, therefore, in absolute measures, produced higher levels of each of the respective IgG subclasses. Also, mice receiving CLP formulated with MP produced higher proportions of IgG1 (nonsignificant trend). Mice receiving the CLP vaccine with AlOH produced distinctly higher IgG1 levels (Figure 3a, *p* = 0.02 compared to no adjuvant) as well as a markedly lower IgG2a levels (nonsignificant trend) in comparison to the unadjuvanted CLP group.

### 3.3. Comparison of IgG Responses Induced by Prototype CLP Vaccine Delivered via Different Routes of Immunization

**Figure 4:**
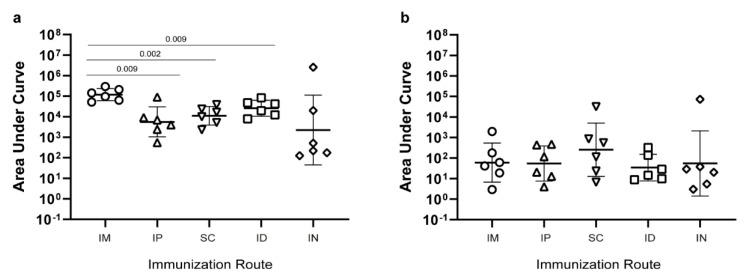
Immunogenicity of the SpyCatcher-AP205-L2 CLP vaccine using different immunization routes. Anti-SpyCatcher IgG (**a**) and anti-HPV L2 peptide IgG (**b**) levels were measured as the area under the curve (AUC) (i.e. optical density (O.D.) at 450 nm multiplied by the dilution factor). Groups of mice (n = 6) were immunized with an equal dose of the unadjuvanted SpyCatcher-AP205-L2 CLP vaccine administered via different immunization routes: intramuscular (IM), intradermal (ID), subcutaneous (SC), intraperitoneal (IP), and intranasal (IN). Kruskal–Wallis test showed that there was a statistically significant difference between two or more groups for SpyCatcher, *p* = 0.01 (panel (**a**)), but not for HPV peptide, *p* = 0.68 (panel (**b**)). For SpyCatcher, uncorrected, pairwise Mann–Whitney nonparametric tests were used to compare the IM immunized groups with the other routes of immunization. Significant differences are indicated in the graph.

To investigate the immunogenicity after delivering the un-adjuvanted prototype CLP vaccines using different routes. A total of 2 µg of the CLP vaccine was immunized intramuscularly (IM), subcutaneously (SC), intradermally (ID), or intranasally (IN) in 30 µL volume as well as 100 µL intraperitoneally (IP). IM vaccination elicited the highest geometric mean IgG levels (measured as the area under the curve (AUC), corresponding to 1–2 log higher endpoint IgG titers (Supplementary Figure S3). However, robust anti-SpyCatcher IgG levels were also found in mice vaccinated by the other routes (Figure 4a). In all animals, the levels of anti-HPV peptide IgG levels were markedly lower following all routes of immunization (Figure 4b), and the previously observed advantage of IM immunization was no longer apparent.

## 4. Discussion

The ultimate goal of vaccination against infectious diseases is to raise long-lived, protective immune responses with as few administrations and side-effects as possible. CLPs have gained increasing interest as vaccine vehicles as they share many characteristics with live-attenuated vaccines that are capable of inducing long-lived protective immunity after a single immunization [52]. The modular approach for covalent attachment of antigens to AP205 CLP using split-protein technology has established itself as a potent and versatile vaccine platform [14]. To further characterize the platform, this study measured humoral responses to a prototype AP205 CLP vaccine (displaying a protein and a peptide antigen) with different extrinsic adjuvants and immunization routes.

An important confirmation was that CLP display could dramatically improve the immunogenicity of a protein antigen (SpyCatcher), whereas formulation of the same antigen with extrinsic adjuvants alone had minimal effects. Similar results were previously reported for other antigens (e.g., the malaria protein Pfs25 [14]) showing that CLP display can activate different or additional immune mechanisms compared to the tested extrinsic adjuvants.

Interestingly, formulation of the prototype AP205 CLP vaccine with different extrinsic adjuvants was capable of either increasing or decreasing the immunogenicity of the CLP-displayed antigens. Specifically, formulation with LMQ, SWE, and MPL increased antibody responses, whereas formulation with CL, AlOH, and MP resulted in similar (AIOH/CL) or decreased (MP) antibody levels compared to immunization with AP205 CLP alone.

A particle size range of 20–200 nm allows for draining to secondary lymphoid organs [4,5]. By contrast, large micron-sized particles are dependent on dendritic cells (DCs) for transport to the draining lymph node and typically remain at the site of injection [53]. It is possible that lymph node drainage of the AP205 CLP was prevented by adsorption on MP, which offers an explanation for the reduced endpoint antibody responses of the AP205 CLP/MP vaccine formulation. The LMQ and MPL adjuvants increased the IgG elicited by the CLP vaccine. Both adjuvants include a TLR-4 agonist (corresponding to monophosphoryl lipid A [28]); a possible explanation could be that auxiliary immune stimulation of the B cell, through engagement of TLR4 together with rigid, high-density, and oriented antigen display could cause the observed synergistic increase in antigen-specific antibody levels. The question remains whether this observation can be translated to the clinic, since TLR4 is constitutively expressed on murine B cells but not in human [54]. However, clinical studies comparing the immunogenicity of different HPV CLP formulations, containing either AlOH or AlOH plus the TLR4 agonist, revealed that the latter formulation elicited higher levels of neutralizing antibodies as well as a higher frequency of memory B cells [55,56]. Comparable results have also been reported for the enveloped Hepatitis B surface Ag virus-like particle (VLP) vaccine [57]. Recombinant AP205 CLPs (and other bacteriophage CLPs) contain encapsulated host RNA from their production in *E. coli*. The bacterial RNA significantly boosts the immunogenicity of the CLP via TLR7/8 activation [58]. Furthermore, multiple studies have demonstrated that co-delivery of TLR4 and TLR7/8 agonists potently and synergistically enhances antigen-specific immune responses [59,60,61]. This is evidenced by increased cytokine secretion leading to intensified germinal center formation, including antibody class switching [59,60,61]. Accordingly, the increased immunogenicity observed by formulation of the prototype AP205 CLP vaccine with LMQ or MPL alone is likely caused by the described synergistic interplay between the TLR-4 and TLR7/8 activation pathways. A related example of adjuvants working in synergy is documented by studies on AS01, which shares similar immune potentiators as those included in the LMQ adjuvant (MPL-A and QS21) [26,62]. These studies showed that a novel IFNgamma-related pathway was engaged only when both immune-modulating components were present and that this pathway appears crucial for optimal activation of DCs as well as Th1 response induction [26,62]. However, since immunizing with SWE also increases the overall immunogenicity, the observation could also simply be an additive adjuvant effect. Future experiments could decipher TLR involvement by immunizing TLR knockout mice.

The relative production of IgG antibody subclasses after vaccination can act as an indication of the type of elicited immune response. In mice, production of the IgG1 subclass is correlated with having more engaged Th2 type responses whereas the production of IgG2a, IgG2b, and IgG3 is more indicative of a Th1 bias [63,64,65].

For many vaccines (e.g., against intracellular pathogens), it would be necessary to induce cellular immune responses [66]. The intrinsic features of CLP make them very efficient at raising both humoral and cellular immunity [67,68]. Host RNA encapsulated in the lumen of *E. coli*-produced bacteriophage CLPs further promotes cellular immunity through Th1-type responses (evidenced by antibody class switching to IgG2 and IgG3 subclass in mice) via TLR7/8 activation [69,70].

This study shows the AP205 CLP vaccine platform produces both IgG1 and IgG2 subclass antibodies, suggestive of a mixed Th1 and Th2-type response, which confirms previous findings using this platform [22]. Overall, adding extrinsic adjuvants to the CLP vaccine platform does not appear to affect the relative proportions of IgG subclasses. While extrinsic adjuvants such as LMQ, SWE, and MPL did not affect the overall proportions of IgG subclasses, it is important to emphasize that these adjuvants were capable of raising the overall immunogenicity of the antigen and thus amplifying the production of the IgG1 and IgG2 subclasses in terms of absolute antibody amounts.

AlOH (and to some extent MP) stand out, since they can skew the antibody production towards increased proportions of IgG1 and, with respect to AlOH, lower proportions of IgG2a, indicative of an increase in Th2-type responses. This result may be expected as AlOH is known to elicit Th2-type responses characterized by an increased production of Th2 cytokines (IL-4 and IL-13) and transcription factors, which leads to the attenuation of Th1-type responses and Ig class switch to IgG1 and IgE [71,72]. In general, caution needs to be taken when interpreting the IgG subclass results, as vaccine-induced responses are partly species- and even mouse strain-specific [73]. In line with this, the mouse strain used in this study, Balb/c, is known to have an intrinsic Th2 polarized immune response [73]. Additionally, this study looks exclusively at the humoral immune response induced after immunization. While the ratio of IgG subclasses can be used as surrogate markers, future studies should look further into how the CLP vaccines activate the cellular arm of the immune system and how this may be affected by the use of different adjuvants.

When comparing the elicited IgG responses to the CLP vaccine administered without an adjuvant, we found that IM immunizations resulted in the highest levels of anti-SpyCatcher IgG whereas the IgG response against the HPV L2 peptide was similar across the different immunization routes. In general, systematic studies investigating the immunological effects of using different immunization routes is lacking. Today, most vaccines used in humans, including the licensed VLP-based vaccines, are administered IM. However, the preference for IM over SC is not based on clinical data showing improved immune responses after IM delivery [74]. Similarly, mouse studies testing different immunization routes for VLP vaccines show no clear immunological advantage of using IM over SC delivery. A specific study using simian-human immunodeficiency virus-like particles provided evidence to suggest that intradermal vaccination may be superior to other routes of immunization [5], but it is unclear to what extent the presented data was antigen-specific. Finally, a previous study testing SC versus IN delivery of another bacteriophage-derived CLP (Qbeta) showed that delivery via the IN route resulted in induction of the mucosal IgA and IgG whereas SC immunization could only elicit mucosal IgG. Therefore, in cases were a mucosal IgA response is essential, there may be an actual need for the mucosal immunization route [75].

## 5. Conclusions

The results highlight that the AP205 CLP platform by itself is efficient at inducing high antibody levels with an IgG subclass distribution that is sought after in many indications. Certain routes of immunization and extrinsic adjuvants can further boost immunogenicity either through synergistic interplay or an additive adjuvant effect. Further work is needed to elucidate and optimize CLP vaccine-induced cellular immune responses. Finally, it is of utmost importance to test if modular CLP vaccines can generate high, fast, durable, and immune-tolerance breaking antibody responses in humans, as seen in preclinical studies.

## Figures and Tables

**Table 1:** Overview of the extrinsic adjuvants used in present study.

Adjuvant	Full Name	Composition	Proposed Th1 /Th2 Phenotype	Expected Mode of Action (Based on Comparable Adjuvant Systems)
**LMQ**	Liposomes	Liposomes containing DOPC/cholesterol with TLR4 ligand and QS21 saponin	Th1 [26,27]	TLR4 signaling [28]
	TLR4 ligand			immune cell recruitment, activation, and priming [29]
	saponin			Inflammasome activation and antigen cross presentation (QS21) [30]
**SWE**	Squalene-in-water	Oil-in-water emulsion containing squalene, Tween 80, and Span 85	Th1 and Th2 [31]	Immune cell recruitment [32]
	emulsion			Induction of ATP release [33] causing NALP3 inflammasome-independent release of IL33 [34] and triggering Myd88-dependent signaling [35]
**MPL**	Monophosphoryl	Monophosphoryl	Th1 [36]	TLR4 signaling [28]
	lipid A	lipid A		
**CL**	Cationic	DPPC (1,2-dipalmitoyl-sn-glycero-3-phosphocholine)/DC-cholesterol (3ß-[N-(N’,N’-dimethylaminoethane)-carbamoyl]cholesterol hydrochloride)	Dependent on size [37], charge [38], and rigidity [39]	Antigen delivery and retention [40]
	liposomes			Stimulation/activation of antigen presenting- and epithelial cells [41]
**AlOH**	Aluminum hydroxide	Aluminum	Th2 [42]	Antigen delivery and uptake [43]; immune cell recruitment [44]
				Uric acid [45], DNA [46], and heat-shock protein 70 [47]
		hydroxide		
				NALP3 inflammasome induction [48]
**MP**	Cationic microparticles	PLGA (poly (DL-lactide-co-glycolide)/CTAB (cetyl trimethylammonium bromide)	Th1 and Th2 [49]	Antigen delivery [49], retention, and uptake [50]

**Table 2:** Formulation protocol for the capsid-like particle (CLP) vaccine in adjuvants.

Name	Adjuvant Suspension		Antigen Solution	Injection Volume
	Adjuvant	PBS (µL)	(µL)	(µL)
SWE	175 µL Squalene Water Emulsion	35	140	50
AlOH	61.8 µL Alhydrogel	148.2	140	50
LMQ	70 µL Liposomes		140	50
	70 µL MPL-A			
	70 µL QS21			
CL	70 µL Cationic Liposomes	140	140	50
MP	175 µL Microparticles	35	140	50
MPL	70 µL MPL-A	140	140	50

**Table 3:** Amount of compounds used in the extrinsic adjuvant formulations.

Formulation	µg per Dose of 50 µL
SWE	squalene 1000 µg
AlOH	AlOH 90 µg
LMQ	MPL: 10 µg
	QS21: 10 µg
CL	DPPC: 46 µg
	DC-Chol: 34 µg
Microparticles	PLGA: 250 µg
	Dextran: 125 µg
TLR4 bacterial source	MPL: 10 µg

## Data Availability

All relevant data are accessible within the manuscript and its Supplementary Materials.

## Supplementary Materials

The following are available online at https://www.mdpi.com/2076-393X/9/2/131/s1, Figure S1: Immunogenicity of antigens on CLP in extrinsic adjuvant formulations, Figure S2: Statistical comparisons, and Figure S3: Route of Immunization.
