# Supplementary material for: The Immunogenicity of Capsid-Like Particle Vaccines in Combination with Different Adjuvants Using Different Routes of Administration
Source: Vaccines (Basel). 2021 Feb 6;9(2):131. doi: 10.3390/vaccines9020131 (PMC7915698; doi:10.3390/vaccines9020131)
Supplement: Supplementary file 1 [file vaccines-09-00131-s001.pdf]

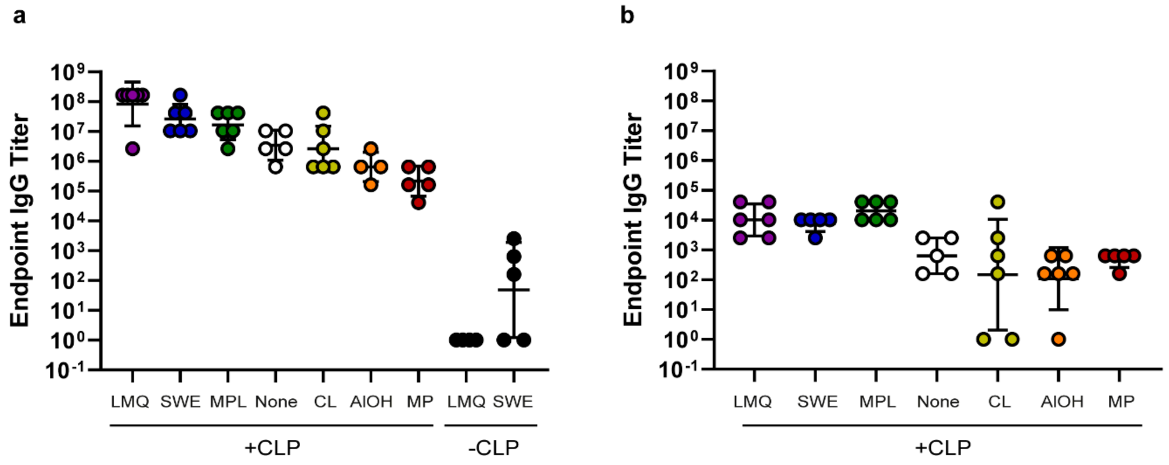

Figure S1. Immunogenicity of antigens on CLP in extrinsic adjuvant formulations: (**a**) SpyCatcher and (**b**) HPV peptide-specific IgG titers following immunization with the SpyCatcher-AP205-L2 (+CLP) vaccine formulated in different adjuvants. Specifically, mice were immunized with liposomes/MPL/QS21 (LMQ, purple, n = 6), squalene water emulsion (SWE, blue, n = 6), monophosphoryl lipid A (MPL, green, n = 6), no adjuvants (none, white, n = 5), cationic liposomes (CL, yellow, n = 6), aluminum hydroxide (AlOH, orange, n = 4), or microparticles (MP, red, n = 5). The control groups are equimolar soluble SpyCatcher proteins formulated in either LMQ or SWE (−CLP, black, n = 6 for both groups). Each circle represents the titer obtained in a single mouse in one representative ELISA assay measurement at an O.D. 450 nm cutoff of 0.1. The geometric mean is shown together with the geometric standard deviation.

a

IgG ELISA (SpyCatcher)	LMQ	SWE	MPL	No adjuvant	CL	AlOH	Microparticles	LMQ control	SWE control
LMQ									
SWE	0.11								
MPL	0.048*	0.73							
No adjuvant	0.01*	0.04*	0.07						
CL	0.009*	0.048	0.10	0.68					
AlOH	0.01*	0.005*	0.02*	0.10	0.28				
Microparticles	0.004*	0.002*	0.004*	0.02*	0.04*	0.29			
LMQ control	0.005*	0.005*	0.005*	0.016*	0.005*	0.03*	0.016*		
SWE control	0.005*	0.002*	0.004*	0.008*	0.002*	0.02*	0.008*	0.17	
**Kruskal-Wallis test**	P<0.0001								

b

IgG ELISA (L2)	LMQ	SWE	MPL	No adjuvant	CL	AlOH	Microparticles
LMQ							
SWE	0.96						
MPL	0.45	0.12					
No adjuvant	0.02*	0.02*	0.002*				
CL	0.06	0.08	0.03*	0.73			
AlOH	0.002*	0.002*	0.002*	0.21	0.8		
Microparticles	0.002*	0.008*	0.002*	>0.99	0.77	0.21	
**Kruskal-Wallis test**	P=0.0003						

c

%IgG1 ELISA (SpyCatcher)	LMQ	SWE	MPL	No adjuvant	CL	AlOH	Microparticles
LMQ							
SWE	0.13						
MPL	0.59	0.04*					
No adjuvant	>0.99	0.26	0.55				
CL	0.92	0.09	0.49	0.71			
AlOH	0.004*	0.002*	0.009*	0.024*	0.002*		
Microparticles	0.1	0.02*	0.1	0.1	0.02*	0.71	
**Kruskal-Wallis test**	P=0.0016						

d

%IgG2a ELISA (SpyCatcher)	LMQ	SWE	MPL	No adjuvant	CL	AlOH	Microparticles
LMQ							
SWE	0.70						
MPL	0.04*	0.09					
No adjuvant	0.26	0.26	>0.99				
CL	0.06	0.06	0.70	>0.99			
AlOH	0.002*	0.002*	0.009*	0.10	0.03*		
Microparticles	0.38	0.55	0.55	>0.99	0.90	0.26	
**Kruskal-Wallis test**	P=0.0085						

e

%IgG2b ELISA (SpyCatcher)	LMQ	SWE	MPL	No adjuvant	CL	AlOH	Microparticles
LMQ							
SWE	0.03*						
MPL	0.24	0.18					
No adjuvant	0.17	0.91	0.55				
CL	0.03*	0.49	0.09	0.55			
AlOH	0.004*	0.13	0.03*	0.26	0.59		
Microparticles	0.26	0.55	0.55	0.70	0.90	0.90	
**Kruskal-Wallis test**	P=0.0380						

f

%IgG3 ELISA (SpyCatcher)	LMQ	SWE	MPL	No adjuvant	CL	AlOH	Microparticles
LMQ							
SWE	0.03*						
MPL	0.31	0.40					
No adjuvant	0.10	0.90	0.26				
CL	0.004*	0.02*	0.06	0.10			
AlOH	0.009*	0.18	0.08	0.39	0.93		
Microparticles	0.02*	0.02*	0.17	0.10	0.90	>0.99	
**Kruskal-Wallis test**	P=0.0052						

g

IgG ELISA (SpyCatcher) Route of Immunization	IM	ID	SC	IP	IN
IM					
ID	0.10				
SC	0.01*	0.21			
IP	0.007*	0.1	0.77		
IN	0.002*	0.007*	0.01*	0.06	
**Kruskal-Wallis test**	P=0.0005				

h

IgG ELISA (L2) Route of Immunization	IM	ID	SC	IP	IN
IM					
ID	0.03*				
SC	0.05	0.92			
IP	0.18	0.47	0.71		
IN	0.02*	>0.99	>0.99	0.71	
**Kruskal-Wallis test**	P=0.0005				

Figure S2. Statistical comparisons: Mann–Whitney nonparametric tests were used for all tests of significance included in this paper. Statistic differences are highlighted with an asterix (*).

- a- Anti-SpyCatcher IgG ELISA (corresponding to Figure 2a).
- b- Anti HPV IgG ELISA (corresponding to Figure 2b).
- c- Relative IgG1 titers (corresponding to Figure 3a).
- d- Relative IgG2a titers (corresponding to Figure 3b).
- e- Relative IgG2b titers (corresponding to Figure 3c).
- f- Relative IgG3 titers (corresponding to Figure 3d).
- g- Anti-Spycatcher IgG titers following different immunization routes (corresponding to Figure 4a).
- h- Anti-HPV IgG titers following different immunization routes (corresponding to Figure 4b).

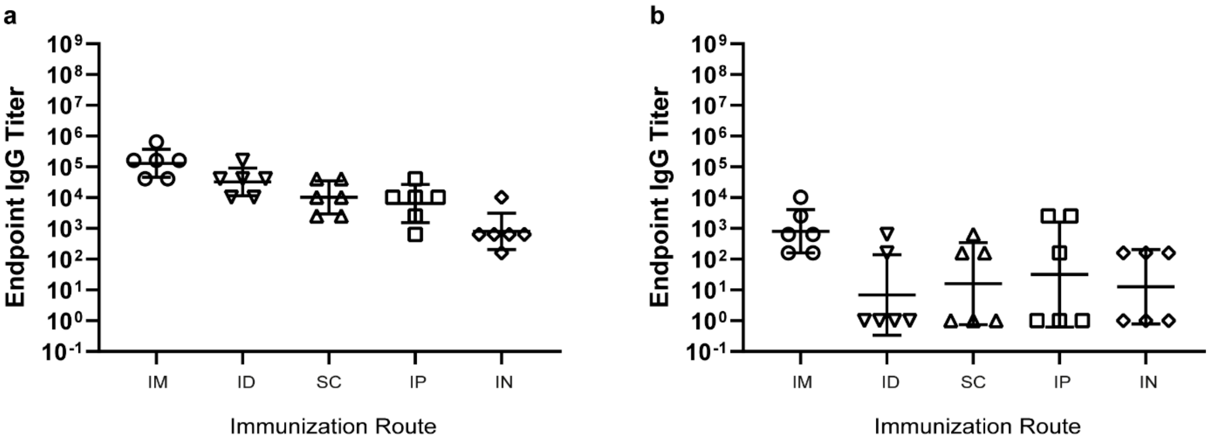

Figure S3. Route of Immunization: the characterization of immunogenicity following administration of the CLP vaccine via different routes of immunization. (**a**) SpyCatcher and (**b**) HPV peptide-specific endpoint IgG titers were established at an O.D. 450 nm cutoff of 0.1. All mice (n = 6 per immunization route) received unadjuvanted SpyCatcher-AP205-L2. The immunization routes were intramuscular (IM), intradermal (ID), subcutaneous (SC), intraperitoneal (IP), and intranasal (IN). The geometric mean is shown together with geometric standard deviation.
